# Application of real-time reverse transcription polymerase chain reaction to the detection the matrix, H5 and H7 genes of avian influenza viruses in field samples from South Korea

**DOI:** 10.1186/1743-422X-10-85

**Published:** 2013-03-14

**Authors:** Hye-Ryoung Kim, Jae-Ku Oem, You-Chan Bae, Min-Su Kang, Hee-Soo Lee, Yong-Kuk Kwon

**Affiliations:** 1 Animal, Plant and Fisheries Quarantine and Inspection Agency, 175 Anyangro, Manangu, Anyangsi, Gyeonggido 430-757, South Korea

**Keywords:** Avian influenza virus, Real-time reverse transcription polymerase chain reaction (RRT-PCR), H5 and H7 subtype, Virus detection

## Abstract

**Background:**

The rapid and accurate identification of the H5 and H7 subtypes of avian influenza (AI) virus is an important step for the control and eradication of highly pathogenic AI outbreaks and for the surveillance of AI viruses that have the potential to undergo changes in pathogenicity in poultry and wild birds. Currently, real-time reverse transcription polymerase chain reaction (RRT-PCR) is routinely used for the rapid detection of the H5 and H7 genes, but misidentification is frequent for emergent isolates and viruses isolated from diverse regions due to the high sequence variation among AI viruses.

**Findings:**

In this study, an RRT-PCR method was tested for the detection of matrix, H5 and H7 genes from diverse subtypes of AI viruses and from field samples obtained through AI surveillance in South Korea over the last four years. Both RRT-PCR and conventional experiment (virus isolation using egg inoculation followed by reverse transcription polymerase chain reaction) agreed on the virus-positive samples. And the comparison of the results with 174 clinical samples showed a high level of agreement without decreasing the specificity and sensitivity.

**Conclusions:**

This assay could be useful tool for the rapid detection of AI using the field samples from domestic poultry and wild birds in South Korea, and continuous regional updates is needed to validate primer sets as the AI virus evolves.

## Findings

Influenza A viruses are classified on the basis of the antigenic properties of their surface glycoproteins hemagglutinin (HA) and neuraminidase (NA). Influenza A has been demonstrated to have 16 HA subtype and 9 NA subtypes, and viruses of all subtypes and viruses of the majority of possible combinations have been isolated from avian species [1,2].

Influenza A viruses containing the HAs of subtypes H5 and H7 may become highly pathogenic upon introduction into poultry, and highly pathogenic avian influenza (HPAI) viruses causing devastating economic losses in the poultry industry have been found to belong to the H5 or H7 subtype [3-6]. Therefore, the ability to rapidly characterize AI viruses is crucial for facilitating the timely implementation of control measures [7].

Real-time reverse transcription polymerase chain reaction (RRT-PCR) assays have been developed and are used globally: these assays are used for the rapid detection of H5 in many Asian countries (H5N1 epidemic) and as a screening method in several countries in Europe and North America [8-11]. The evaluation and design of primer and probe sets are key elements in establishing a method to identify AI subtypes due to the high sequence variation among AI viruses of diverse origins and the emergence of new isolates [12,13].

Virus isolation (VI) using egg inoculation and conventional reverse transcription polymerase chain reaction (RT-PCR) has been conducted for the diagnosis of AI in South Korea since the first HPAI outbreak in 2003. However, the establishment of RRT-PCR as a first-line screening method to process high numbers of samples is important because of the increasing need for AI surveillance studies in wild birds and domestic ducks, which are hypothesized to be strong candidates for the introduction of HPAI and to be reassortment vessels [14,15].

A 10% homogenate of feces or cloacal swabs was prepared in phosphate-buffered saline and centrifuged, and the supernatant was used to inoculate the embryonating chicken eggs (ECEs). Viruses tested were propagated in 9- to 11- day-old ECEs and harvested from allantoic fluids of inoculated eggs after 4 days of incubation at 37°C. Hemagglutination (HA) test was practiced with the collected allantoic fluids using 1% chicken red blood cell. Viral RNA was extracted from HA positive-allantoic fluids using Viral Gene-spin™ Viral DNA/RNA extraction kit (iNtRON biotechnology, Inc., South Korea). AI viruses were subtyped using RT-PCR tests that were performed using the *AccuPower*® RT-PCR premix (Bioneer, Daejeon, South Korea) amplification reagents and the primer sets from previously published reports [16-19] by using the following temperature profile: one cycle of 30 min at 42°C and 5 min at 94°C, 40 cycles that each consisted of 30-s at 94°C, 30-s at 57°C and 30-s at 72°C, and extension at 72°C for 10 min. The RT-PCR products were run on a 1% agarose gel stained with ethidium bromide and electrophoresed at 100 V for 20 min.

For new RRT-PCR test, viral RNA was extracted from the supernatant of field samples using the same kit mentioned above. And all RRT-PCR assays were performed with a one-step PrimeScript™ RT-PCR kit (TaKaRa, Kyoto, Japan) in a 25-μl master reaction mixture containing the following components: 12.5 μl of kit-supplied 2X One-Step RT-PCR buffer III, 0.5 μl of kit-supplied TaKaRa Ex Taq™ HS, 0.5 μl of kit-supplied PrimeScript™ RT enzyme Mix II, 4 μl of RNA template and 0.5 μl each of forward and reverse primer (20 pmol), 0.5 μl of probe (6 pmol) and sufficient RNase-free water to bring the final volume to 25 μl. RRT-PCR assays were performed in a Smart Cycler II thermocycler with the following thermocycling conditions: a 5-min reverse transcription step at 42°C, a 10-sec heat activation of the Taq polymerase at 95°C a 40 cycles of PCR amplification. The conditions for each PCR varied depending on the target gene as follows: for the matrix gene, we used a 1-s denaturation at 95°C and a 20-s step annealing at 60°C; for the H5 subtype gene, we used a 1-s denaturation at 95°C, a 20-s annealing step at 57°C and a 5-s e1ongation step at 72°C; and for the H7 subtype gene, we used a 1-s denaturation step at 95°C, a 20-s annealing step at 45°C and a 5-s step e1ongation at 72°C.

Previous published forward and reverse primers and probes for the matrix and H5 genes [20,21] were observed the concern for sensitivity to detect low pathogenic avian influenza (LPAI) viruses including H5 subtype isolated in South Korea. Based on sequence comparison including 40 LPAI H5 gene, 70 HPAI H5 genes and Korean AI viruses genes [GenBank: JX235977 to JX236026, JX258651 and JX258652], the primer and probe sets were used intact or were modified to contain 1 or 2 mixed bases (Table 1 and Additional file 1: Table S2). The primer and probe sets for the H7 genes were designed in this study using sequences (37 genes from Eurasian-lineage viruses and 20 genes from South Korea strains) downloaded from the Influenza Virus (Table 1). Because of the significant sequence variability of H7 genes, multiple alignment was performed and primers and probe were designed to minimize nucleotide mismatches in the Eurasian H7 viruses (data not shown). All probes were labeled at the 5^′^ end with 6-carboxyfluorescein (FAM) (reporter dye) and at the 3^′^ end with the 6- carboxytetramethylrhodamine (TAMRA) (quencher dye).

**Table 1:** Primers and probes for detecting of matix, H5 and H7 genes of avian influenza viruses

**Gene**	**Primer or probe**	**Sequence**	**Product size (bp)**	**Reference**
Matrix	M-Kr forward	AAGACCAATCCTGTCACCTCTGA	104	[21]
	M-Kr reverse	CAAAGCGTCTACGCTGCAGTCC		[21]
	M-Kr probe	FAM-TTTGTNTTYACGCTCACCGTGCC-TAMRA		modified [21]
H5	H5-Kr forward	TGACTACCCGCAGTATTCAG	145	[20]
	H5-Kr reverse	AGACCAGCTAYCATGATTGC		modified [20]
	H5-Kr probe	FAM-TCAACAGTGGCRAGTTCCYTAGCA-TAMRA		modified [20]
H7	H7-Kr forward	ATAGCRGGTTTYATTGAAAA	134	newly designed
	H7-Kr reverse	CCTGTTATTTGATCAATTGC		newly designed
	H7-Kr probe	FAM-TGGGAAGGTCTVRTTGAYGG-TAMRA		newly designed

The 50% egg infectious dose (EID_50_) titers were determined in 11-day-old SPF ECEs, and virus titers were calculated by the Reed and Muench method [22]. To calculate the limit of detection of RRT-PCR method, RNAs from A/duck/Korea/Cheonan/2010(H5N1) of 7.8 logEID_50_/0.1 ml titer and A/duck/Korea/A76/2010(H7N7) of 6.8 logEID_50_/0.1 ml titer were extracted and serially diluted 10-fold in molecular biology-grade water and tested with Table 1 primer sets.

The sensitivity and specificity of the RRT-PCR assay were compared with those of VI in embryonating eggs combined with RT-PCR using 15 reference influenza viruses with different HA types, 71 isolates of Korean AI viruses, and 174 fecal or cloacal samples collected during the HPAI outbreak season from 2010 to 2011 in South Korea.

The primer and probe set was tested with RNA obtained from influenza virus isolates representing all 15 HA subtypes of human- and avian-origin influenza viruses and 71 avian influenza viruses with various subtypes isolated from South Korea. The matrix primer set was able to detect most of the type A influenza viruses. The RRT-PCR for the matrix gene detected 98.8% of 86 influenza A viruses of various subtypes and origins and showed high sensitivity (Table 2 and Additional file 1: Table S1). The A/duck/Czechoslovakia/1956(H4N6) virus was not detected by the matrix primer set; the sequence of this old H4N6 strain has one mismatch each with the reverse primer and the probe in this set.

**Table 2:** Real-time RT-PCR application to avian influenza viruses^* ^**isolated in South Korea (No. of positive result/No. of tested viruses)**

	**H1**	**H2**	**H3**	**H4**	**H5**	**H6**	**H7**	**H9**	**H10**	**H11**	**H12**	**Total**
M	6/6	3/3	9/9	8/8	10/10	6/6	8/8	8/8	6/6	5/5	2/2	71/71
H5	-^†^	-	-	-	9/10^‡^	-	-	-	-	-	-	9/10
H7	-	-	-	-	-	-	8/8	-	-	-	-	8/8

^*^List of Korean AI viruses was shown in Additional file 1: Table S2.

^†^Not test.

^‡^A/wildbird/L60-2/08(H5N2) virus was not detected.

The H5 and H7 primer sets detected the RNAs from the virus isolates of their respective subtypes. The RRT-PCR assays for H5 and H7 showed positive results for most of LPAI viruses of the H5 and H7 subtypes and for two H5N1 HPAI viruses isolated in 2006 and 2010 in South Korea, but the A/wild bird/L60-2/2008(H5N2) virus was not detected (Table 2 and Additional file 1: Table S1). The A/wild bird/L60-2/2008(H5N2) virus is known to exhibit an unusual sequence relative to other H5N2 LPAI viruses isolated in South Korea [23,24].

A total of 174 clinical samples were collected from 58 chickens, 64 domestic ducks and 52 wild birds with suspected HPAI infections. A total of 101 samples of the 174 samples were positive for type A influenza virus by both VI plus RT-PCR and RRT-PCR. Sixty-nine samples were negative for type A influenza virus by both methods. Four samples were positive by RRT-PCR but negative by VI plus RT-PCR. Seventy-nine H5 samples and 2 H7 samples were positive in both assays, and 95 and 172 samples, respectively, were negative in both assays (Table 3). The results of the VI plus RT-PCR assay and the RRT-PCR assay for detecting the H5 and H7 genes did not differ, but the two assays did not agree with respect to the matrix gene for four samples. These samples were collected from one domestic duck and three mallards and the results of the RRT-PCR assay for the matrix gene showed high Ct values (> 38), that were close to the cutoff of 40 cycles to be considered negative.

**Table 3:** Summary of real-time RT-PCR and virus isolation/RT-PCR results for 174 individual samples from poultry and wild birds

	**Current method**	**New method (real time RT-PCR)**	
	**(VI/RT-PCR)**	**Positive**	**Negative**
	**(No. of samples)**		
M	positive (101)	101	0
	Negative (73)	4	69
H5	positive (79)	79	0
	negative (95)	0	95
H7	positive (2)	2	0
	negative (172)	0	172

Therefore, the sensitivity of this new method assay to detecting the M, H5 and H7 genes of AI viruses were 99.5% (186/187), 98.9% (89/90) and 100% (11/11) and the specificity of that were 94.5% (69/73), 100% (170/170) and 100% (249/249), respectively.

The average detection limits (50% egg infective doses [EID_50_]/0.1 ml) of the matrix, H5 and H7 genes were 10^1.8^, 10^2.8^ and 10^1.8^ EID_50_/0.1 ml, respectively.

Since the 2008 H5N1 HPAI outbreak in South Korea, hundreds of thousands of samples have been collected every year from wild birds in migratory bird habitats, from domestic ducks on farms and from poultry at live bird markets for the detection AI viruses to control the spread of HPAI [14,25]. However, VI plus RT-PCR protocol, the standard method for AI surveillance, is time and labor intensive and there is a possibility of cross-contamination when handling the infectious samples. Therefore, we applied and evaluated the RRT-PCR as a screening method for AI virus detection and subtyping using the clinical samples. This assay showed high sensitivity and specificity in the evaluation of AI viruses of diverse origins and various HA types and in the evaluation of clinical samples, relative to the VI plus RT-PCR assay.

However, the test failed to detect the matrix gene of the A/duck/Czechoslovakia/1956(H4N6) virus and the H5 gene of the A/wild bird/L60-2/2008(H5N2) virus, although one or two mismatches are tolerable. The reason for this failure is unclear, but it may be due to the mismatch in the reverse primer with both viruses and not to the mismatches in the probes [26]. There were false positive results for the matrix gene for four fecal samples from three wild birds and a domestic duck. The high Ct values were near the cutoff for a negative result, and additional studies must be performed to determine the best method to remove RRT-PCR inhibitors associated with fecal samples.

Reports of the validation of any test generally specify the species and type of sample tested [27]. Our study was performed using diverse AI viruses isolated in South Korea from 2008 to 2011 and clinical samples from poultry and wild birds collected during the H5N1 HPAI outbreak from 2010 to 2011. Moreover, the isolation of many AI viruses, sequencing analysis and AI virus characterization in South Korea enabled us to apply and evaluate this RRT-PCR assay [23,25,28-31].

This RRT-PCR test described in the present study could be a useful diagnostic tool for rapid screening and surveillance in wild birds and poultry. The identification of the H5 and H7 genes for the rapid detection of HPAI is important as a control measure of outbreak. And the detection of the M gene of influenza A virus from the field samples of wild birds and poultry is also necessary for the further study like virus isolation and characterization of AI viruses, as a few known pandemics in humans and several outbreaks in poultry have had their origins in LPAI viruses of other subtypes [32].

Finally, the regularly re-evaluation of this assay is essential to use as the official tool for AI surveillance in South Korea, because the HA gene of Influenza A virus is well known for its high sequence divergence both between and within subtypes [33] and newly evolving emergent viruses could be introduced from other countries H5N1 HPAI endemic.

## Competing interests

The authors declare that they have no competing interests.

## Authors’ contributions

HRK carried out the evaluation and design of the primer sets, participated in the sequence alignment and drafted the manuscript. JKO and YCB participated in sample collection, virus isolation and real-time RT-PCR. MSK and HSL conducted data analysis and helped the design of the study. YKK participated in its design and coordination and helped to draft the manuscript. All authors read and approved the final manuscript.

## Supplementary Material

Additional file 1: Table S1 Real-time RT-PCR application to reference avian influenza virus examined in this study. **Table S2.** Korean avian influenza viruses examined in this study. Click here for file
