# Supplementary material for: Application of real-time reverse transcription polymerase chain reaction to the detection the matrix, H5 and H7 genes of avian influenza viruses in field samples from South Korea
Source: Virol J. 2013 Mar 14;10:85. doi: 10.1186/1743-422X-10-85 (PMC3606358; doi:10.1186/1743-422X-10-85)
Supplement: Additional file 1: Table S1 — Real-time RT-PCR application to reference avian influenza virus examined in this study. Table S2. Korean avian influenza viruses examined in this study. [file 1743-422X-10-85-S1.doc]

Additional file

Table S1. Real-time RT-PCR application to reference avian influenza virus examined in this study

| ***Strain*** | **M** | **H5** | **H7** |
| --- | --- | --- | --- |
| A/PuertoRico/8/34 (H1N1) | + | - | - |
| A/Singapore/1/57 (H2N2) | + | - | - |
| A/duck/Ukrine/1/63 (H3N8) | + | - | - |
| A/duck/Czechoslovakia/56 (H4N6) | - | - | - |
| A/duck/Hongkong/820/80 (H5N3) | + | + | - |
| A/Shearwater/Australia/1/72 (H6N5) | + | - | - |
| A/duck/Hongkong/301/78 (H7N1) | + | - | + |
| A/turkey/Ontario/6118/68(H8N4) | + | - | - |
| A/turkey/Wisonsin/1/66 (H9N2) | + | - | - |
| A/chicken/Germany/N/49 (H10N7) | + | - | - |
| A/duck/England/56 (H11N6) | + | - | - |
| A/duck/Alberta/60/76 (H12N5) | + | - | - |
| A/gull/Maryland/704/77 (H13N6) | + | - | - |
| A/mallard/Gurjev/263/82 (H14N5) | + | - | - |
| A/Shearwater/w.Australia/2576/79 (H15N6) | + | - | - |

Table S2. Korean avian influenza viruses examined in this study

| Subtype | Isolate | Genbank accession number(M/HA^*^) |  | Subtype | Isolate | Genbank accession number(M/HA) |
| --- | --- | --- | --- | --- | --- | --- |
| H1 | Dk/Kr/70-1/08(H1N3)  Wb/Kr/A07/09(H1N3)  Wb/Kr/A392/09(H1N1)  Wb/Kr/A01/10(H1N3)  Wb/Kr/A45/10(H1N1)  Wb/Kr/A19/11(H1N1) | JX235999  JX236012  JX236016  JX236011  JX236017  JX236014 |  | H6 | Dk/Kr/334-15/08(H6N1)  Dk/Kr/A39/08(H6N2)  Dk/Kr/A127/09(H6N2)  Dk/Kr/A377/09(H6N2)  Dk/Kr/A23/11(H6N2)  Dk/Kr/A74/11(H6N2) | GQ414931  GQ414928  GQ414929  JX235998  JX235989  JX236003 |
| H2 | Wb/Kr/L94-1/08(H2N9) | JX236010 |  | H7 | Wb/Kr/A330/09(H7N7) | JN244122 / JN244227 |
|  | Wb/Kr/L112-1/08(H2N3) | JX236021 |  |  | pintail/Kr/1173/09(H7N7) | JN244123 / JN244229 |
|  | mallard/Kr/1216/10(H2N3) | JX236006 |  |  | mallard/Kr/822/10(H7N7) | JN244125 / JN244230 |
| H3 | Dk/Kr/L57-6/08(H3N2) | JX236005 |  |  | Wb/Kr/A72/10(H7N2) | JN244124 / JN244247 |
|  | Dk/Kr/A78/08(H3N2) | JX235977 |  |  | Dk/Kr/A75/10(H7N7) | JN244139 / JN244239 |
|  | Dk/Kr/A120/09(H3N2) | JX235978 |  |  | Dk/Kr/A76/10(H7N7) | JN244130 / JN244245 |
|  | Dk/Kr/A37/10(H3N2) | JX236000 |  |  | Dk/Kr/A117/10(H7N6) | JN244132 / JN244238 |
|  | Dk/Kr/A54/10(H3N2) | JX235984 |  |  | Wb/Kr/A84/11(H7N7) | JX235994 / JX236024 |
|  | Dk/Kr/A90/10(H3N2) | JX235986 |  | H9 | Ck/Kr/L37-4/08(H9N2) | JX235995 |
|  | Dk/Kr/A21/11(H3N2) | JX235988 |  |  | Dk/Kr/A70/08(H9N2) | JX235997 |
|  | Dk/Kr/A80/11(H3N8) | JX235992 |  |  | Ck/Kr/A33/09(H9N2) | JX235982 |
|  | Dk/Kr/A83/11(H3N2) | JX236004 |  |  | Dk/Kr/A174/09(H9N2) | GU086242 |
| H4 | Dk/Kr/A57/08(H4N6) | JX236002 |  |  | Ck/Kr/A229/09(H9N2) | JX235980 |
|  | Wb/Kr/A222/09(H4N6) | JX235979 |  |  | Dk/Kr/A60/10(H9N2) | JX235985 |
|  | Dk/Kr/A255/09(H4N3) | JX235981 |  |  | Ck/Kr/A22/11(H9N2) | JX235996 |
|  | Wb/Kr/A17/10(H4N6) | JX235983 |  |  | Dk/Kr/A65/11(H9N2) | JX235991 |
|  | Wb/Kr/A46/10(H4N6)  Wb/Kr/A15/11(H4N6)  Dk/Kr/A57/11(H4N2)  Dk/Kr/A82/11(H4N6) | JX236018  JX235987  JX235990  JX235993 |  | H10 | Wb/Kr/L110-2/08(H10N4) | JN817537 |
|  |  |  |  |  | pintail/Kr/188/09(H10N4) | JN817539 |
|  |  |  |  |  | Wb/Kr/A01/09(H10N6) | JN817535 |
|  |  |  |  |  | Wb/Kr/A43/09(H10N4) | JN817536 |
| H5 | Ck/Kr/IS/06(H5N1) | EU233676 / EU233675 |  |  | Wb/Kr/A13/10(H10N1) | JN817543 |
|  | Dk/Kr/A14/08(H5N2) | GU086237 / GU086230 |  |  | mallard/Kr/1203/10(H10N8) | JN817533 |
|  | Wb/Kr/L60-2/08(H5N2) | GU086239 / GU086229 |  | H11 | Wb/Kr/L86-1/08(H11N9) | JX236022 |
|  | Dk/Kr/A93/08(H5N2) | GU086238 / GU086231 |  |  | Wb/Kr/A67/09(H11N9) | JX236019 |
|  | Wb/Kr/A81/09(H5N2) | GU086240 / GU086232 |  |  | Wb/Kr/A164/09(H11N9) | JX236013 |
|  | Wb/Kr/A278/09(H5N2) | JX236007 / JX236025 |  |  | Wb/Kr/A343/09(H11N9) | JX236008 |
|  | Wb/Kr/A344-2/09(H5N1) | JX236015 / JX258651 |  | H12 | Dk/Kr/A42/08(H12N5) | JX236001 |
|  | Dk/Kr/Cheonan/10(H5N1) | JN808063 / JN807985 |  |  | Wb/Kr/L86-3/08(H12N2) | JX236023 |
|  | Wb/Kr/A71/10(H5N2) | JX236020 / JX258652 |  |  |  |  |
|  | Wb/Kr/A51/11(H5N3) | JX236009 / JX236026 |  |  |  |  |

* HA gene: H5 and H7 subtype virus
